# A case of esophageal atresia with the bronchial-like lower esophagus which originates from the left lower lobe bronchus

**DOI:** 10.1186/s40792-022-01513-7

**Published:** 2022-08-15

**Authors:** Terutaka Tanimoto, Takuo Noda, Reisuke Imaji, Hiroshi Nouso

**Affiliations:** 1 Department of Pediatric Surgery, Okayama University Hospital, 2-5-1 Shikatacho, Kita-ku, Okayama, Okayama 700-8558 Japan; 2 Department of Pediatric Surgery, Hiroshima City Hiroshima Citizens Hospital, 7-33 Motomachi, Naka-ku, Hiroshima, Hiroshima 730-8518 Japan

**Keywords:** Esophageal atresia, Communicating bronchopulmonary foregut malformation, Broncho-esophageal fistula

## Abstract

**Background:**

Esophageal atresia with or without a trachea–esophageal fistula occurs due to the failure of separation or incomplete development of the foregut. Therefore, esophageal atresia is often associated with various forms of tracheobronchial anomalies. We report an extremely rare case of esophageal atresia.

**Case presentation:**

A female infant was born at 37 weeks of gestation and weighed 2596 g. A diagnosis of esophageal atresia and total anomalous pulmonary vein return type III were confirmed. The infant had respiratory distress that required tracheal intubation and ventilatory support soon after birth. Temporary banding of the gastroesophageal junction and gastrostomy were performed on the second day of life. However, her respiratory condition deteriorated due to atelectasis of the left lung and compensatory hyperinflation of the right lung. Preoperative examinations showed the unilobe and atelectatic left lung. The trachea was trifurcated in three directions, and the branch that was expected to be the left main bronchus was blind-ended. The dorsal branch was cartilaginous and bifurcated into the left lower lobe bronchus and lower esophagus approximately 1 cm distal from the tracheal trifurcation. The cartilaginous tissue continued to the lower esophagus. The diagnosis of esophageal atresia with the lower esophagus which originated from the left lower lobe bronchus was made. Esophageal atresia repair was performed when the patient was 4 months of age. The esophagus was dissected distally to the bifurcation of the left lower lobe bronchus via right thoracotomy. The lower esophagus was bronchial-like in appearance, transitioning to the normal esophageal wall approximately 7 mm distal to the transected edge. The cartilage tissue was completely resected during surgery, and a primary end-to-end anastomosis of the esophagus was successfully performed. Histopathological findings revealed that the extracted specimen was surrounded by tracheal cartilage and that the inner surface was covered by stratified squamous epithelium that originated from the esophagus.

**Conclusions:**

In cases of esophageal atresia with an atypical clinical presentation, there may be unique structural abnormalities of the foregut. We emphasize the importance of a preoperative surgical planning since an inadequate operation can lead to fatal complications.

## Background

Esophageal atresia (EA) with or without a trachea–esophageal fistula (TEF) occurs due to the failure of separation or incomplete development of the foregut [1]. Therefore, EA is often associated with various forms of tracheobronchial anomalies. We present an extremely rare case of EA in which the lower esophagus originated from the left lower lobe bronchus and transitioned from the bronchial tissue to esophageal tissue.

## Case presentation

**Fig. 1:**
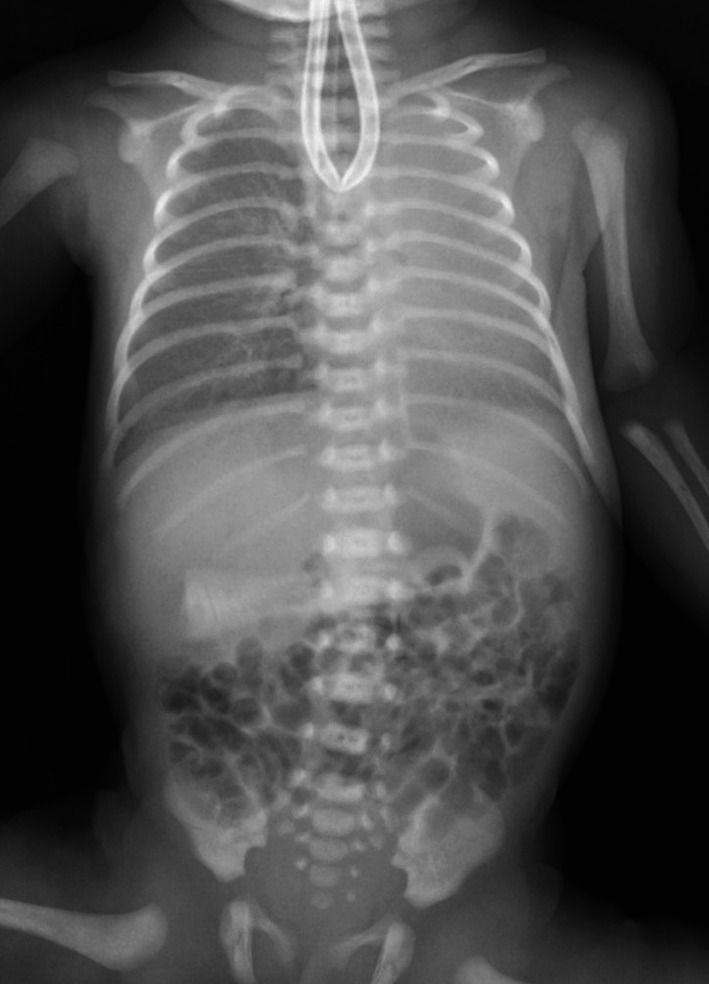
Chest radiography taken after birth showing folding of the nasogastric tube in the upper pouch and hypoplasia of the left lung

A female infant was born at 37 weeks of gestation and weighed 2596 g. A diagnosis of EA and total anomalous pulmonary vein return type III (TAPVR type III) were confirmed by chest radiography and ultrasonography, respectively (Fig. 1). The infant had respiratory distress that required tracheal intubation and ventilatory support soon after birth. Temporary banding of the gastroesophageal junction and gastrostomy were performed on the second day of life. However, her respiratory condition deteriorated due to atelectasis of the left lung and compensatory hyperinflation of the right lung. Because of this atypical clinical course, she was transferred to our hospital.

**Fig. 2:**
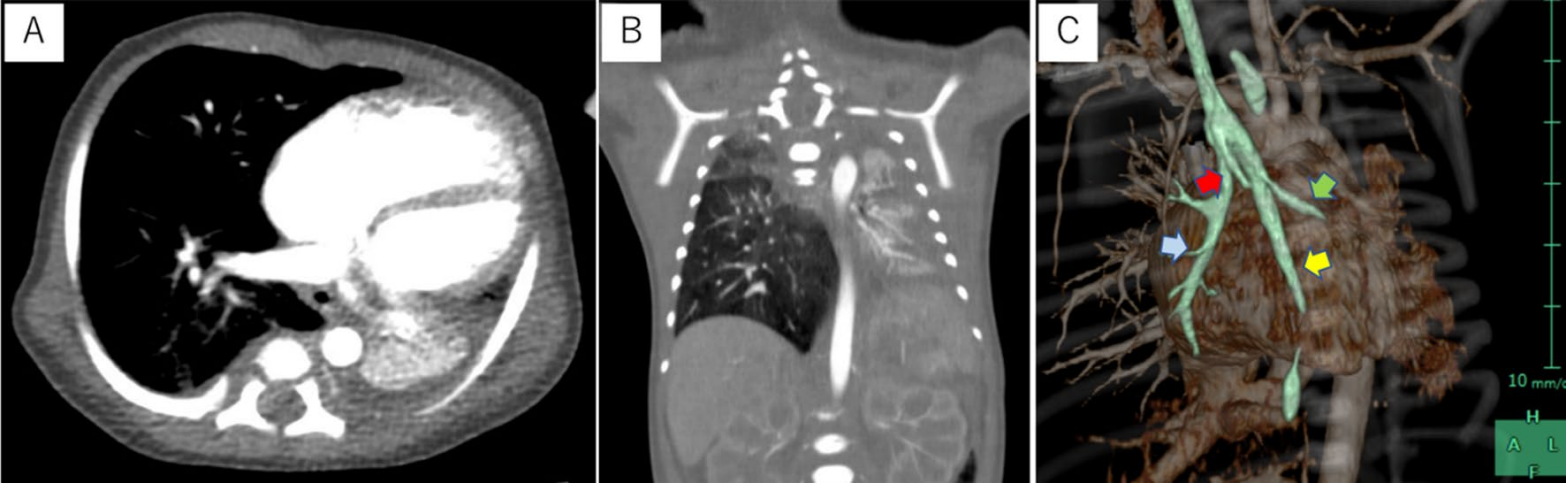
Contrast-enhanced computed tomography (CT) showing an atelectatic unilobe in the left lung (**A**, **B**). Three-dimensional CT image representing the tracheal trifurcation in three directions; the right main bronchus (blue arrow), blind-ended branch considered to be the left main bronchus (red arrow), and dorsal branch bifurcated to left lower lobe bronchus (green arrow) and the lower esophagus (yellow) (**C**)

Contrast-enhanced computed tomography (CT) showed an atelectatic unilobe in the left lung. The trachea was trifurcated in three directions. The morphology of the right main bronchus was normal, and the branch that was expected to be the left main bronchus was blind-ended. The dorsal branch was bifurcated into the left lower lobe bronchus and lower esophagus approximately 1 cm distal from the tracheal trifurcation (Fig. 2). Based on these findings, communicating bronchopulmonary foregut malformation (cBPFM) Group IA, an EA combined with an affected lung bronchus originating from the lower esophagus, was suspected.

**Fig. 3:**
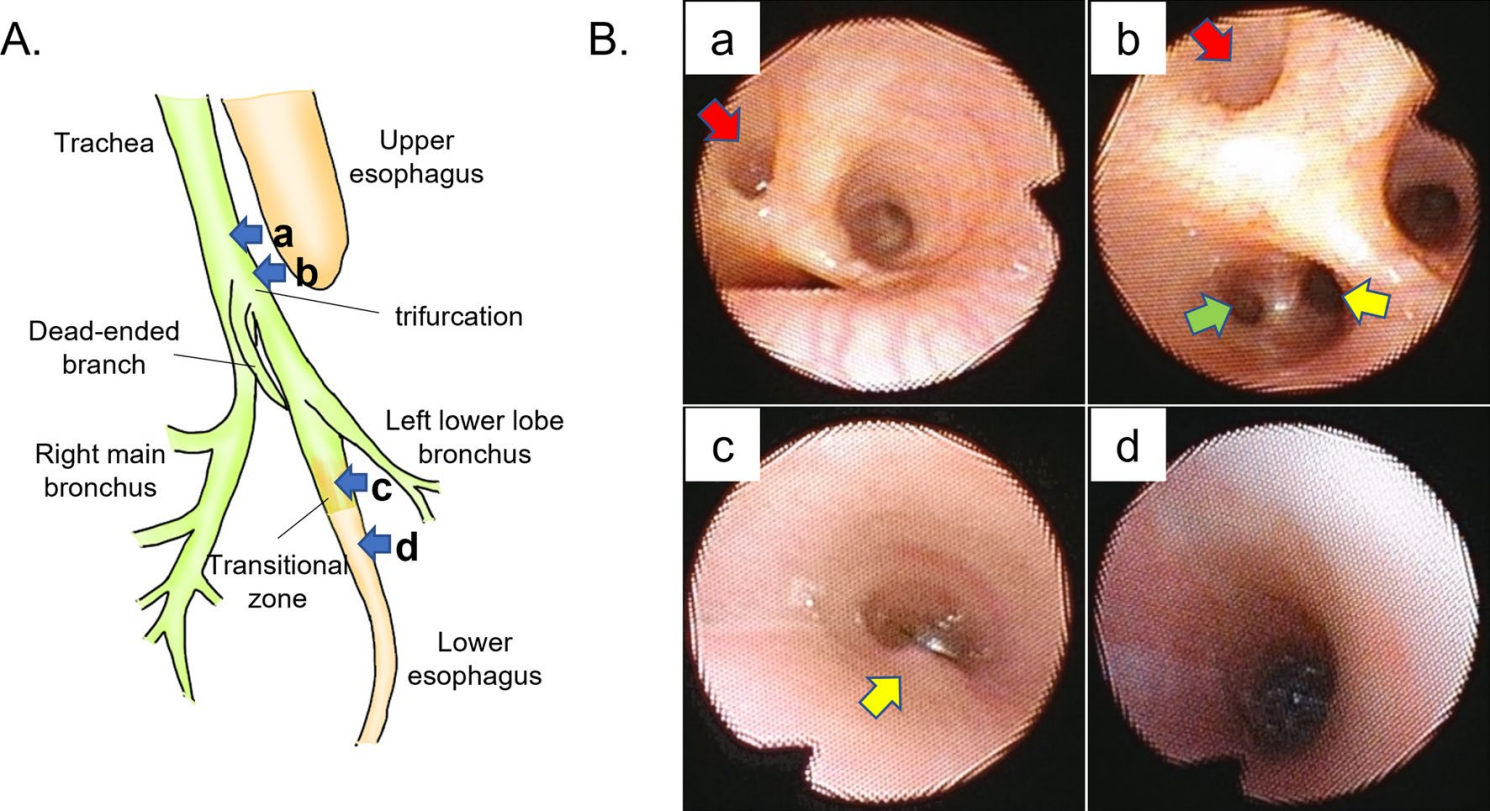
**A** The scheme represents the airway (green) and esophagus (yellow). The lowercase letters in the figure correspond to each bronchoscopy finding in **B**. **B** The trachea was trifurcated in three directions. The left branch was blind-ended (red arrow) (**a**). The dorsal branch was cartilaginous and bifurcated into the left inferior lobe bronchus (green arrow) and lower esophagus (yellow arrow) (**b**). Cartilage tissue was observed in the lower esophagus, presenting a bronchial-like appearance (**c**). A normal esophageal wall appeared distally (**d**)

In cases of cBPFM Group IA, the esophagus proximal to the bifurcation of the left lower lobe bronchus would be presumed to be easily collapsed without positive pressure ventilation. However, the tracheobronchoscopy examination revealed that the dorsal branch was cartilaginous, and the lumen’s shape was retained without positive pressure (Fig. 3). Furthermore, cartilage tissue was also observed in the lower esophagus, presenting a bronchial-like appearance that transitioned to a normal esophageal wall in the middle of the lower esophagus. These findings suggested that this was not a case of cBPFM Group IA, but was instead type C EA where the lower esophagus originated from the left lower lobe bronchus. This meant that air entering into the left lower lobe would be maintained after the dissection of the esophagus distal to this bifurcation. We performed TAPVR repair when the patient was 3 months of age and planned the EA repair when the patient was 4 months of age.

Right thoracotomy with a transpleural approach was applied to minimize the compression of the right lung. After carefully defining the bifurcation of the left lower lobe bronchus and lower esophagus by bronchoscope observation, the esophagus was dissected a few millimeters distal to the bifurcation. The lower esophagus was bronchial-like in appearance, transitioning to the normal esophageal wall approximately 7 mm distal to the transected edge. To avoid postoperative complications of esophageal stricture, the cartilaginous esophagus was completely removed. Approximately 10 mm of the lower esophagus was resected, resulting in a larger distance between the upper and lower esophagus than predicted. By performing Livaditis myotomy, the esophagus could be primarily anastomosed.

**Fig. 4:**
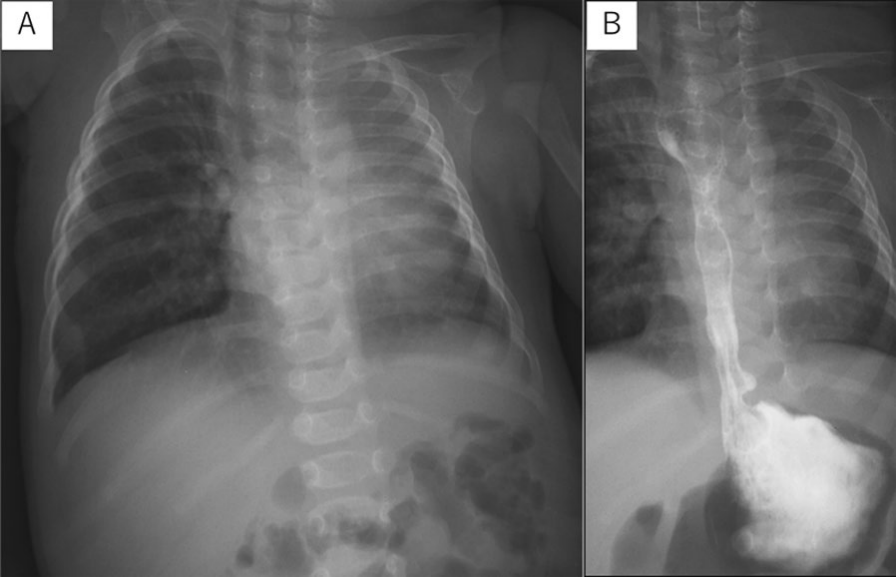
Postoperative chest radiography showing a well aerated left lung (**A**). Esophagography showing satisfactory passage at the anastomotic site (**B**)

**Fig. 5:**
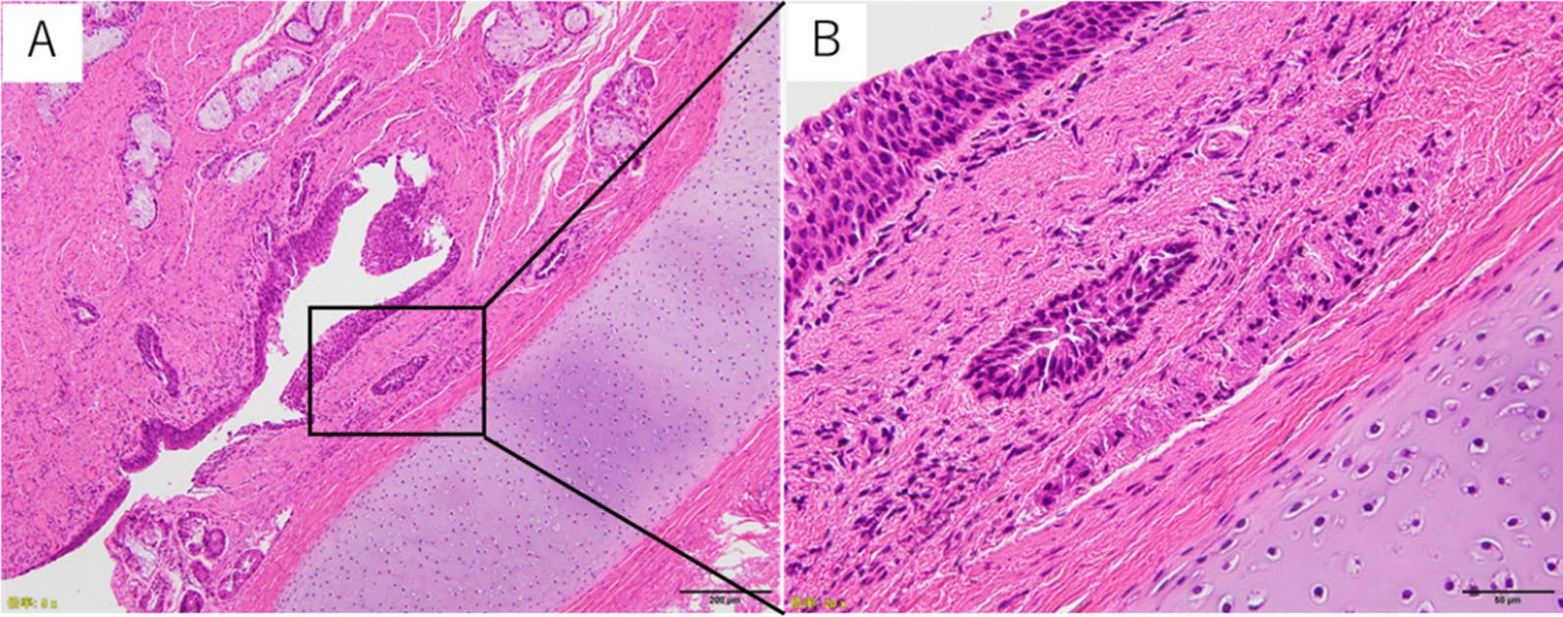
Histological findings of the resected specimen showing the tracheal cartilage and the stratified squamous epithelium which originated from the esophagus. (**A** H&E × 100, **B** H&E × 400)

Postoperatively, the left lung was well aerated, and the baby was extubated on postoperative day 5. Anastomotic stricture, possibly due to postoperative scarring, required 2 dilatations, however, the passage of food then became satisfactory (Fig. 4). Histopathological findings revealed that the resected specimen was surrounded by tracheal cartilage, and the mucosa layer included a stratified squamous epithelium that originated from the esophagus (Fig. 5). These findings demonstrated that this specimen included the transitional area from the bronchus to the esophagus.

## Discussion

The etiology of EA with or without TEF is reported to occur due to the failure of separation or incomplete development of the foregut. Therefore, EA is often comorbid with various forms of tracheobronchial anomalies [2]. cBPFM, one of these malformations, is defined by a patent congenital communication between the esophagus or stomach and an isolated portion of the respiratory tract [3]. Srikanth et al. [4] classified cBPFM into four groups. Among these, group IA is associated with EA/TEF and the entire lung arises from the lower esophagus or stomach. The corresponding mainstem bronchus is absent from the trachea.

cBPFM has diagnostic difficulties [5], and in our case, the challenge was to differentiate between a diagnosis of cBPFM type IA and an atypical case of type C EA. In general, a case of type C EA has the entry site of the lower esophagus located around the tracheal bifurcation, in which case the esophagus would need to be dissected at this site and an end-to-end esophageal anastomosis is performed. In the case of cBPFM group IA, although the lower esophagus needs to be dissected and anastomosed at the same site as in type C EA, tracheobronchial anastomosis [6] or lobectomy [7] of the esophageal-originating lung is additionally needed. Our case was different from both cases. In our patient, the malformation formed as a type C EA with an additional characteristic that the bronchial-like lumen was observed from tracheal trifurcation to the lower esophagus. Therefore, it was necessary to dissect the lower esophagus distal to this bifurcation and to resect the bronchial-like portion of the lower esophagus. In each of these cases, the surgical procedures are completely different, and inadequate operations can cause fatal complications. A preoperative surgical planning is crucial in cases of EA with an atypical clinical course.

Regarding the diagnosis of this case, there is an alternative explanation. We diagnosed this case as esophageal atresia with the bronchial-like lower esophagus which originates from the left lower lobe bronchus. However, this case could also be considered as a case of cBPFM Group IA with bronchial-like lower esophagus. The main difference between these diagnoses is whether the dorsal branch of tracheal trifurcation is considered as a left lower lobe bronchus or a bronchial-like lower esophagus. Since they present similar morphologies, it is difficult to distinguish between them. This is the first case report of this kind of malformation, making it difficult to classify this case.

The left lung was unilobed, and a left-sided approach with right one-lung ventilation was considered. However, the patient had a usual left-sided aortic arch; hence, a left-sided approach was expected to have a higher risk of intraoperative organ injury and postoperative esophageal obstruction due to flexion. This logic is similar to a case, in which the patient had an EA with a right-sided aortic arch that was treated surgically by a right-sided approach [8, 9]. Therefore, we selected a right-sided approach. Furthermore, a transpleural approach was applied to minimize the compression of the right lung. This was because the thoracoscopic approaches would compress the entire right lung, resulting in intraoperative respiratory distress. In addition, we thought it would be safer to compress the lung under direct observation by transpleural approach rather than by extrapleural approach.

The transected edge of the esophagus contained cartilage around two-thirds of its circumference, presenting as a bronchial-like appearance. It has been reported that small islands of cartilage are present within the lower esophageal wall in some cases of EA [10]. However, there have been no previous reports in which the lower esophagus showed a bronchial-like appearance and transitioned into the esophagus. This case is extremely uncommon among the types of tracheoesophageal malformations. In these types of cases, we recommend complete resection of the bronchial-like tissue at surgery. The remnant of the esophagus wall would certainly cause the same situation as congenital esophageal stenosis [11]. Extensive resection of the lower esophagus can make primary repair of EA/TEF challenging and conversion of the surgical procedure should be taken into consideration.

## Conclusions

We experienced a case of EA that required differential diagnosis from cBPFM type IA. In cases of EA with an atypical clinical presentation, there may be unique structural abnormalities of the foregut. We emphasize the importance of a preoperative surgical planning in EA since an inadequate preoperative diagnosis can lead to fatal complications.

## Data Availability

The dataset supporting the conclusion of this article is included within the article.

## Abbreviations

EA: Esophageal atresia
TEF: Tracheoesophageal fistula
TAPVR: Total anomalous pulmonary vein return
